# Optimum Placement of Heating Tubes in a Multi-Tube Latent Heat Thermal Energy Storage

**DOI:** 10.3390/ma14051232

**Published:** 2021-03-05

**Authors:** Mohammad Ghalambaz, Hayder I. Mohammed, Ali Naghizadeh, Mohammad S. Islam, Obai Younis, Jasim M. Mahdi, Ilia Shojaeinasab Chatroudi, Pouyan Talebizadehsardari

**Affiliations:** 1 Metamaterials for Mechanical, Biomechanical and Multiphysical Applications Research Group, Ton Duc Thang University, Ho Chi Minh City 758307, Vietnam; mohammad.ghalambaz@tdtu.edu.vn; 2 Faculty of Applied Sciences, Ton Duc Thang University, Ho Chi Minh City 758307, Vietnam; 3 Department of Physics, College of Education, University of Garmian, Kurdistan 46021, Iraq; hayder.i.mohammad@garmian.edu.krd; 4 Faculty of Mechanical Engineering, Babol University of Technology, Babol 7116747148, Iran; ali.naghizadeh2412@gmail.com; 5 School of Mechanical and Mechatronic Engineering, Faculty of Engineering and Information Technology, University of Technology Sydney, Ultimo, NSW 2007, Australia; MohammadSaidul.Islam@uts.edu.au; 6 Department of Mechanical Engineering, College of Engineering at Wadi Addwaser, Prince Sattam Bin Abdulaziz University, Wadi Addwaser 11991, Saudi Arabia; oubeytaha@hotmail.com; 7 Department of Mechanical Engineering, Faculty of Engineering, University of Khartoum, Khartoum 11111, Sudan; 8 Department of Energy Engineering, University of Baghdad, Baghdad 10071, Iraq; jasim@siu.edu; 9 Department of Mechanical Engineering, Sirjan University of Technology, Sirjan 7813733385, Iran; i.s.chatroudi@gmail.com

**Keywords:** phase change material, melting, latent heat thermal energy storage, Taguchi method, optimization

## Abstract

Utilizing phase change materials in thermal energy storage systems is commonly considered as an alternative solution for the effective use of energy. This study presents numerical simulations of the charging process for a multitube latent heat thermal energy storage system. A thermal energy storage model, consisting of five tubes of heat transfer fluids, was investigated using Rubitherm phase change material (RT35) as the. The locations of the tubes were optimized by applying the Taguchi method. The thermal behavior of the unit was evaluated by considering the liquid fraction graphs, streamlines, and isotherm contours. The numerical model was first verified compared with existed experimental data from the literature. The outcomes revealed that based on the Taguchi method, the first row of the heat transfer fluid tubes should be located at the lowest possible area while the other tubes should be spread consistently in the enclosure. The charging rate changed by 76% when varying the locations of the tubes in the enclosure to the optimum point. The development of streamlines and free-convection flow circulation was found to impact the system design significantly. The Taguchi method could efficiently assign the optimum design of the system with few simulations. Accordingly, this approach gives the impression of the future design of energy storage systems.

## 1. Introduction

Providing facilities to cover all human needs for abundant on-demand energy leads the total power consumption to increase swiftly. Normal life pushes humans to use energy-driven machines and tools such as cooking, cooling, and heating, food storing [1]. Latent heat thermal energy storage systems (LHTES) with phase change materials (PCMs) provide a solution for the mismatches between energy supplies and demands by offering a more compact storage volume compared with conventional hot water storage tanks [2]. Moreover, this method is valuable during both charging and discharging processes, as phase change temperatures are almost constant which makes it more reliable for domestic applications [3]. This technique is included in solar thermal plants, energy management, peak-shaving, water heat recovery, building heating and cooling, and electronic power management [4]. This is relatively promising for applications needing rigid operation temperatures. Because of the weak thermal conductivity of PCMs, the thermal efficiency of thermal energy storage systems (TES) suffers from low heat transfer rates. Different methods are used to enhance the thermal conductivity, such as utilizing different configurations of fins [5], changing the configuration of the geometry [6,7,8], metal foam [9], and nanotechnology [10,11], using multiple PCMs [12], and using the combinations of different methods [12]. LHTES systems naturally result in a compact thermal energy storage design due to the high energy density of phase change.

Urban areas are one of the main power consumers by utilizing 45% of the total energy usage responsible for huge greenhouse gas emissions [13]. Combining PCMs into energy store units has been extensively studied in the literature [14,15,16,17,18]. The purpose was to shift the required electric power or a portion of it from peak times (high-cost tariff) to off-peak times (low-cost tariff). PCM phase changing delivers an active method to store energy during the night and consume it during the day. In this technique, the relatively low temperature at night is utilized to cool down the storage unit, and then, it could absorb the building’s heat during the day. This decreases the condenser operating temperature and consequently higher efficiency.

Mahdi et al. [19] investigated the thermal improvement methods utilizing static structures, nanomaterials, and the combination of both methods (hybrid technique). Generally, it is found that the hybrid improvement procedures utilizing a heat transfer fluid pipe with a fin or metal foam are the most effective methods. Although each method has its challenges and difficulties in operation, there is no desirable technique, considering the effects of conduction and free convection which must be accounted for in the design process. Yamaha et al. [20] studied the effects of various PCM combinations packed in an air channel as a thermal energy storage component for the air-cooling system of a typical building in Nagoya (Japan). They found that a 5.4 kg PCM per square meter is sufficient to maintain a room temperature for 3 h (13:00–15:00).

Zhao et al. [21] integrated a conventional air cooling system with a TES unit to improve the efficiency of the system. The TES unit was made of a shell and a tube and filled with a PCM. The system operated with water and air as heat transfer fluid (HTF) in both melting and solidification loops, respectively. They stated that the coefficient of performance (COP) increases by 25.6% compared with the conventional air conditioning unit.

Many TES units suffer from low heat transfer properties of PCMs and cannot absorb/release the heat on demand. Thus, a proper design of such systems is the key to reach high efficiency. As a result, many researchers have focused on the influences of design parameters on TES units’ efficiency. Dolado et al. [22] studied the important design variables of an LHTES unit. The results showed that the geometrical characteristics of the PCM layer and the air channels, as well as the HTF flow rate, could control the unit performance. 

Waqas and Kumer [23] investigated a solar unit integrated with a PCM layer to warm a building room. The HTF flow rate, PCM mass, and fusion temperature are the essential design parameters affecting the unit performance. 

Diarce et al. [24] utilized numerical simulations and drove a correlation between the solidification rate and a PCM layer’s thickness. This correlation was suitable for the calculation of the thickness of the PCM layer. Ren et al. [13] assessed the heat transfer efficiency of TES photovoltaic collectors. The authors reported that the fusion temperature, PCM type, and HTF flow rate are the major parameters manipulating the stored thermal energy. Amin et al. [25] designed an optimized unit of TES through a parametric investigation. In this work, an efficient indicator, integrating the thermal efficiency and the power storage density, was adopted as a goal to optimize the PCM design. Saman et al. [26] investigated a combined TES-roof solar system and found that the HTF temperature and the flow rate are the two main factors affecting the PCM’s thermal response of the system.

Developing an optimum technique requires a full study, which covers all cases with different parameters. Therefore, this approach needs a lot of running processes, which takes a long time to study numerical simulation and waste materials for the experimental study. Taguchi method is a technique used to specify the best and optimal cases for running [13,27,28,29,30,31,32]. Wang et al. [27] combined the extension theory Taguchi method (ETM) to reduce the required running cases and optimized thermal system elements such as full cells, hydrogen tanks, and electrolyzers. They compared their optimum design with the literature works and found the ETM is the most accurate among other methods. Sun et al. [28] utilized liquid fraction as an indicator and tested the energy charging cycles using the Taguchi method. Liu et al. [29] studied the energy efficiency of a hybrid unit collection of a PCMs-ventilated Trombe wall and a photovoltaic/heat panel combined with PCM using the Taguchi method. 

The current work presents a design optimization approach for TES units with multiple HTF tubes. Unlike most existing investigations, the optimum design was settled considering the HTF tubes’ locations through the PCM domain to achieve the best location of the tubes. Various simulations were designed using the Taguchi method; each design parameter was divided into four levels. The ultimate objective is to increase the signal/noise (S/N) ratio of the corresponding total stored latent heat and categorize the optimal integration of geometric parameters to offer engineers procedural guidance. The proposed optimization strategy offers a technique to optimally place multiple HTF tubes in TES systems.

## 2. Model and Governing Equations

**Figure 1:**
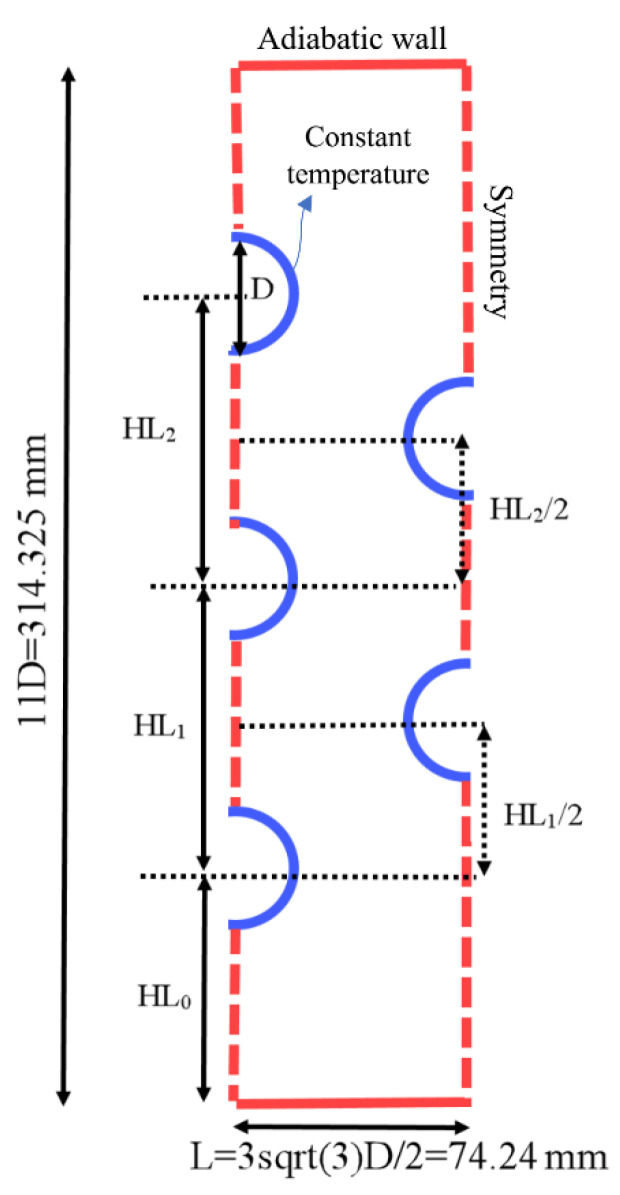
The schematic of the proposed heat exchanger in this study.

Figure 1 illustrates the schematic of the proposed model of the LHTES. The actual LHTES is made of many tubes and could be extended by repeating the pattern in the horizontal direction. Due to the repetition of the inner tubes in the left and right sides of the heat exchanger, the symmetrical boundary condition is applied for the left and right boundaries, denoted by dash lines. The upper and lower walls of the heat exchanger are insulated with no-slip boundary conditions. The temperature of the tubes’ wall is considered constant equal to 50 °C, and the PCM is initially placed at the temperature of 15 °C. It should be noted that the model can be repeated and extended to the full scale of a wide LHTES unit by employing the symmetry boundary conditions indicated by the dashed lines in Figure 1.

As displayed, each tube’s diameter (*D*) is considered constant equal to 25.4 mm (1 inch) with an outer diameter of 28.575 mm. The height of the shell is considered 11D, equal to 314,325 mm, and the width of each repeated section is considered equal to $3\sqrt{3}D/2$ which is equal to 74.24 mm. Three independent parameters (*HL*1, *HL*2, and *HL*3) are defined variables in this study, which determines the tubes’ locations on the left-hand side of the domain. These variables could be optimized to gain the highest melting rate, which is discussed comprehensively in the results and discussion. The locations of the two tubes on the right-hand side of the domain were determined based on the locations of the tubes on the left-hand side of the domain, as illustrated in Figure 1. All the boundary conditions and geometrical variables are included in Figure 1.

For the PCM, RT35 is employed with the mean melting temperature of 32.5 °C, suitable for domestic low-temperature heating applications such as underfloor heating systems. The properties of RT35 are listed in Table 1.

According to the enthalpy-porosity method [34,35], the conservation equations of continuity, momentum, and energy are given considering the following assumptions, i.e., laminar and Newtonian fluid flow for the molten PCM as well as neglecting the viscous dissipation and volume expansion of the PCM during the phase change process. The variation of density is negligible except for the buoyancy force, which is modeled considering the Boussinesq approximation. The gravity, which induces the natural convection flow, is directed downward. Thus, considering the abovementioned assumptions, the governing equations are obtained as following Equations (1)–(3):

(1) $$\frac{\partial \rho }{\partial t}+\nabla \cdot \rho \vec{V}=0,$$

(2) $$\rho \frac{\partial \vec{V}}{\partial t}+\rho \left(\vec{V}\cdot \nabla \right)\vec{V}=-\nabla P+\mu \left(\nabla^{2}\vec{V}\right)-\rho_{ref}\beta \left(T-T_{ref}\right)\vec{g}-A_{m}\frac{{\left(1-\lambda \right)}^{2}}{\lambda^{3}+0.001}\vec{V},$$

(3) $$\frac{\rho C_{p}\partial T}{\partial t}+\nabla \left(\rho C_{p}\vec{V}T\right)=\nabla \left(k\nabla T\right)-\left[\frac{\partial \rho \lambda L_{f}}{\partial t}+\nabla \left(\rho \vec{V}\lambda L_{f}\right)\right].$$

The value 10^5^ was considered for *A_m_* based on the literature and also validation studies [36,37,38]. *λ* was introduced as Equation (4) [39]:

(4) $$\lambda =\frac{\Delta H}{L_{f}}=\left\{\begin{array}{ccc} 0 & if & T<T_{Solidus}\\ 1 & if & T>T_{Liquidus}\\ \frac{T-T_{Solidus}}{T_{Liquidus}-T_{Solidus}} & if & T_{Solidus}<T<T_{Liquidus} \end{array}\right\},$$

where the total enthalpy is the sum of sensitive enthalpy (*h*) and latent heat (Δ*H*) is the volumetric enthalpy (*H*), described as Equations (5) and (6):

*H* = *h* + Δ*H*, (5)

where

(6) $$h=h_{ref}+\int_{T_{ref}}^{T}C_{p}dT,$$

Δ*H* is calculated based on Equation (4) and the molten liquid fraction (*LF*) is the integration of liquid fraction, *λ*, over the computational domain. Moreover, the liquid fraction (*LF*) shows the total amount of the melted PCM divided by the total volume of the phase change material, i.e., the normal total amount of the melted PCM.

## 3. Numerical Method, Mesh Study, and Validation

### 3.1. Numerical Method

In this study, ANSYS workbench software was employed to perform the simulations and geometry generation and also study the effects of different parameters. In this regard, the QUICK scheme was used for the diffusion fluxes and convection, and the PRESTO scheme was used for the pressure equation. The convergence criteria are also considered 10^−4^ for continuity and momentum equations, while 10^−6^ was chosen for the energy equation. 

### 3.2. Mesh and Time-Step Size Study

**Figure 2:**
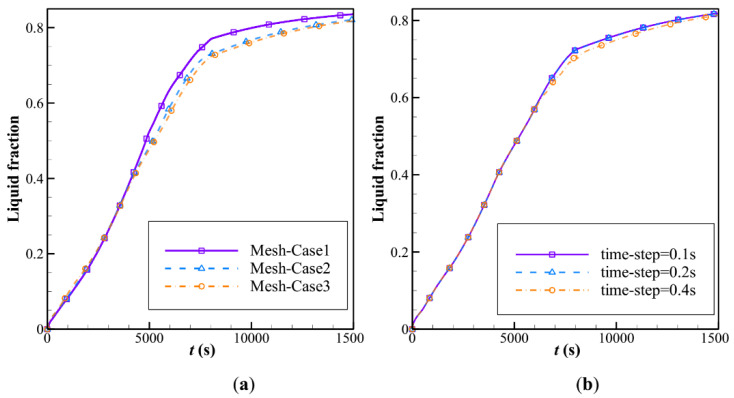
Influence of the mesh size and the time-step on the computed liquid fraction-melting rate (LF) for mesh-case 3 of the L16 table: (**a**) mesh size; (**b**) time-step size for mesh-case 3.

The size of the mesh and the time step of computations can affect the accuracy of computations. Employing a very fine mesh size and small time-steps can increase the computations’ accuracy, but they also increase the demanded computational resources and time. Thus, the fair trade between the accuracy and computational cost can be performed by a mesh study. Here, three mesh sizes were selected, and the computations were repeated for each mesh size. For the mesh study, case 3 from the L16 table was selected for the mesh independence analysis. The computations were executed with a fine time step size of 0.2. The details of mesh sizes and the number of grid points were as follows: mesh-case 1: 24,500; mesh-case 2: 49,000; mesh-case 3: 73,500. The melting rate over time is plotted in Figure 2a. As can be seen, the results for mesh-cases 2 and 3 were very close. Thus, mesh-case 2 was selected for computations. 

The impact of the time-step size on the liquid fraction-melting rate (LF) was studied in Figure 2b over mesh-case 2 for three time-steps of 0.1, 0.2, and 0.4 s. An increase in the time-step enhanced the computational cost significantly, since the governing equations should be solved and converged at each time-step. The results showed that the impact of the time-step on the computed LF was small; however, at an identical time, the liquid fraction varied using 0.4 s as the time-step size compared with that using time-step sizes of 0.2 and 0.1, especially in the middle of the melting process. Thus, the time-step of 0.2 s was selected for all computations of the present research. It should be noted that, after the time-step size analysis, it was concluded that the use of 0.2 s for the time-step size in the mesh independence analysis is correct.

### 3.3. Validation

**Figure 3:**
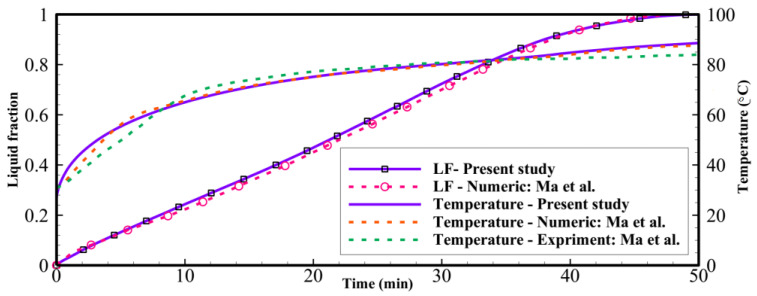
Comparison of the present study with the experimental data of Mat et al. [36].

To verify the simulations in the present study, the experimental and numerical studies of Mat et al. [36] was employed, and the studied geometry was regenerated for the same geometrical and operational parameters. Mat et al. [36] analyzed a double-tube heat exchanger equipped with longitude fins for storing thermal energy. The TES unit in the study of Mat et al. was filled with RT58 as a PCM, and its wall was kept at a uniform temperature. It should be noted that a double-pipe heat exchanger is a simple form of shell-and-tube heat exchangers with one tube inside the shell. Besides, the boundary condition in the study of Mat et al. is a constant wall temperature, which is similar to what is proposed in this study for melting the PCM. Thus, the study of Mat et al. is suitable to verify the code in the present study. The verification results are shown in Figure 3 for both liquid fraction and average temperature presented in this study and those experimental and numerical data reported by Mat et al. [36]. As it can be seen, there is an excellent agreement for further analysis. It should be noted that, in the study of Mat et al., RT58 was employed as a PCM while RT35 was employed for further analysis in this study, since its melting point is suitable for domestic low-temperature heating applications such as underfloor heating systems working as a more efficient system than conventional radiators. 

## 4. Results and Discussion

The Taguchi method was utilized to find the best design of an LHTES unit in the presence of natural convection effects. The buoyancy forces in the molten PCM induced a free convection circulation flow during the charging process. The free convection improved the charging rate by the increase of heat transfer. The free convection heat transfer typically induces a nonuniform melting in an LHTES unit, since the heated liquid tends to move upward and always the top regions of an enclosure are subject to warm currents. Thus, the proper design of energy storage units to fully benefit from natural convection heat transfer is a critical task. Here, the placement height of HTF tubes was adopted as a design parameter. As depicted in schematic Figure 1, there are three geometrical design variables, which are *HL*_0_/*D*, *HL*_1_/*D*, and *HL*_2_/*D*. Following the Taguchi method, each design parameter should be divided into a few possible levels. Here, four levels were considered for each design parameter, of which the details are summarized in Table 2.

The design structure shown in Table 2 was used to construct an orthogonal table and explore the design space. Here, the standard L16 table for three design parameters and four levels was selected. It should be noted that the whole design space could be 4^3^ designs. The Taguchi method aims to find the best design with a minimum computational cost. Thus, instead of 4^3^ simulations, here, only 16 simulations were needed to be executed using the Taguchi method. The computations of PCM melting with convection heat transfer effects were computationally costly. Thus, any reduction in the number of the required simulation is a huge help.

The details of the L16 trial cases are summarized in Table 3. Each row of Table 3 shows an LHTES with a specific placement of the HTF tubes. The target goal for optimization was selected at 75% of the charging process. Thus, a design that could reach a 75% melting volume faster (in a shorter time) is better. Thus, “the smaller, the better approach” was adopted for the Taguchi method.

Each design of Table 3 was simulated, and the required time for 75% melting (*LF* = 0.75) was computed. These data were reported in Table 3. Then, the Taguchi approach was applied to evaluate the value of each design in terms of a characteristic parameter of the S/N ratio.

The relationship for the smaller-is-better *S*/*N* ratio was written using log base [40]:

(7) $$S/N=-10\;\times \;log_{10}\left(\frac{\sum \left(Sa^{2}\right)}{n}\right),$$

where *Sa* denotes the melting time for each test case, and n is the number of observations, which is one in the current research. A design with a large *S*/*N* ratio could lead to a shorter charging time. The computed *S*/*N* ratios are reported in Table 3. Moreover, the *S*/*N* ratios were used to construct Taguchi relations and the ranking table.

Table 4 shows the impact of each design parameter on the charging time of the LHTES unit. Table 4 indicates that the middle right tube (*HL*_1_/*D*) was the most important design parameter and adopted the first rank of significance. After that, the top tube and the bottom tube were the important design parameters.

**Figure 4:**
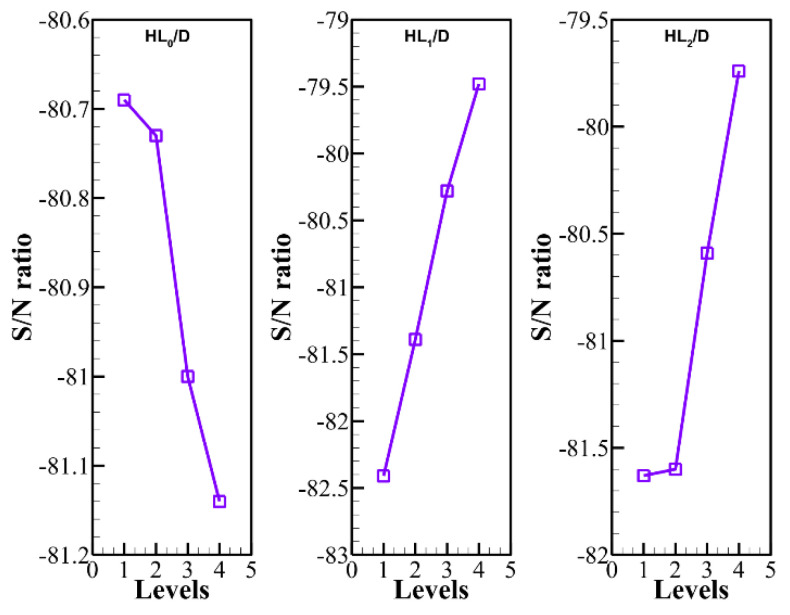
S/N ratios at the levels of the optimum case were as following: *HL*_0_/*D* = 1, *HL*_1_/*D* = 4, and *HL*_2_/*D* = 4.

The results of Table 4 for each parameter and level were collected and shown in Figure 4. Based on the Taguchi method, a design level with a maximum *S*/*N* ratio should be selected as the optimum design level for the parameters. Thus, considering Figure 4, the optimum design corresponded to *HL*_0_/*D* = 1.2, *HL*_1_/*D* = 3.0, and *HL*_2_/*D* = 3.0. The optimum design is depicted in Table 5.

The Taguchi method estimated a thermal charging time of 7915 s. The actual simulations showed a charging time of 8205 s, which was quite close to the estimated value. In general, the optimum case could be a design available in the L16 table or a design out of this table from 4^3^ possible designs. Here, interestingly, the proposed design case was case 4 in the L16 table.

A comparison between the charging times for the optimum case (8205 s) and the worst case of the L16 table (14,472 s) showed that charging time can be changed by 76%. Thus, a simple optimization of the placement of tubes in an LHTES unit could notably improve its performance.

**Figure 5:**
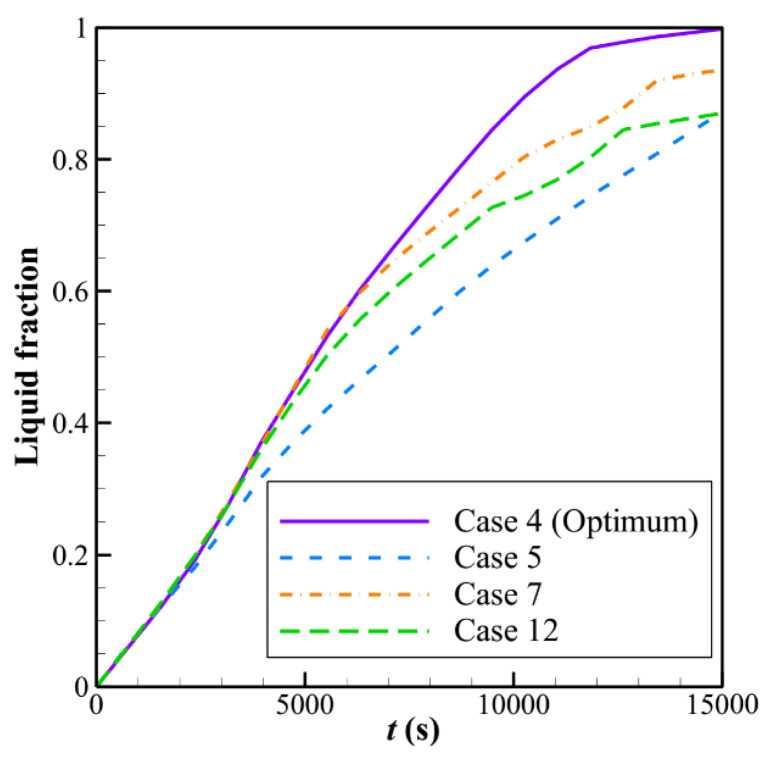
The melting behaviors of the LHTES unit during the time for five selected cases.

Figure 5 illustrates the melting rate (charging) of the LHTES unit during the time. As can be seen, the optimum case quickly reaches high *LF* rates. Interestingly, the behavior of all cases at the beginning of the charging process is almost identical. The reason is that initially, the temperature of the LHTES unit is below the solidus temperature, and thus, the PCM is in the solid phase. Thus, the heat transfer mechanism is solely conduction dominant. The PCM around the tubes act as a large domain. Since the thermal conductivity of PCMs is low, in all design cases, the distance between the tubes is sufficiently large, and they cannot influence each other. After the melting process starts and the melting area extends away from the tubes, the natural convection flows appear. In a convection-dominant flow, the tubes’ locations can significantly control the convection flow and the heat transfer.

**Figure 6:**
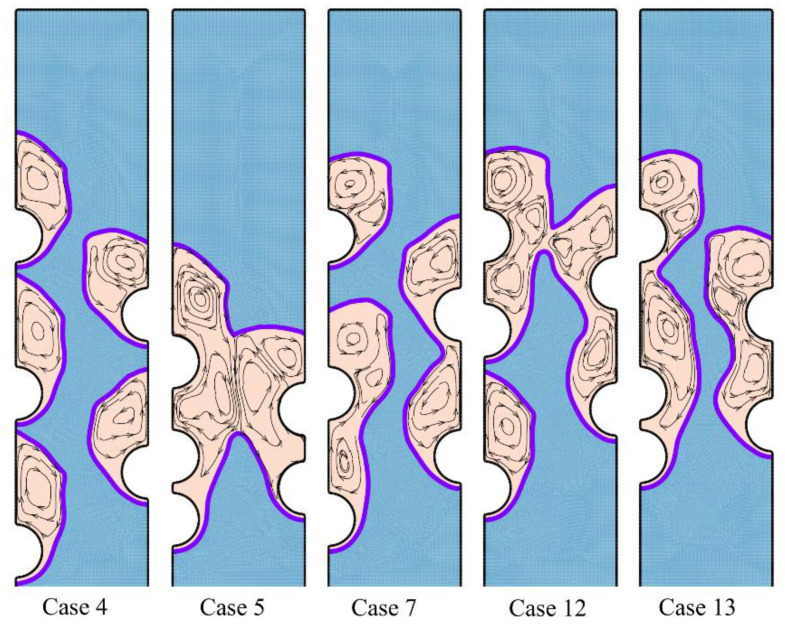
The streamlines and phase change interfaces for five different tubes’ arrangement after one hour of charging. Case 4 is the optimum case with the highest melting rate.

**Figure 7:**
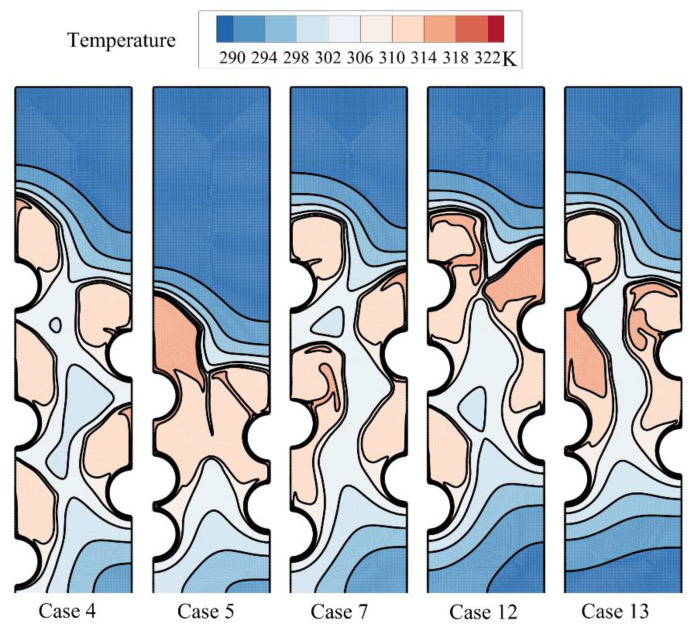
The isotherms for five different arrangements of the tubes after one hour of charging.

**Figure 8:**
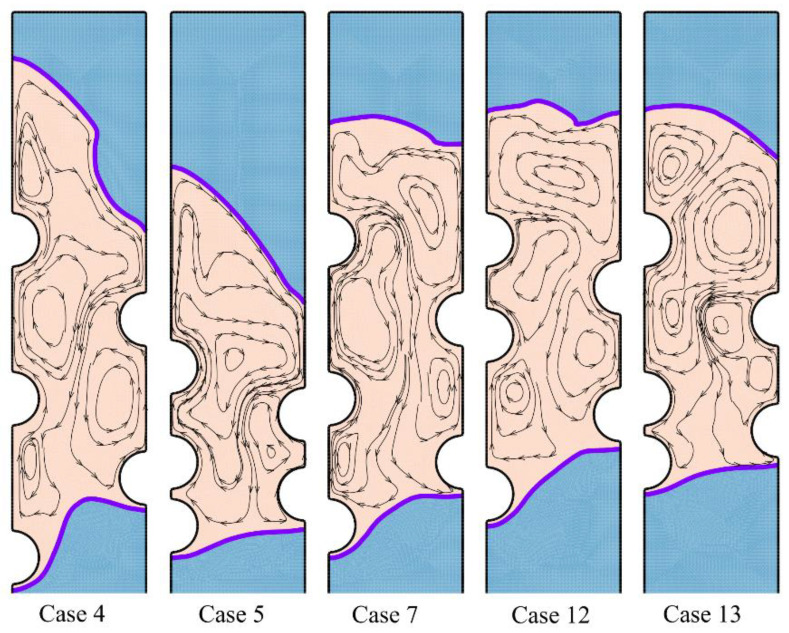
The streamlines and phase change interfaces for five different tubes’ arrangement after two hours of charging.

**Figure 9:**
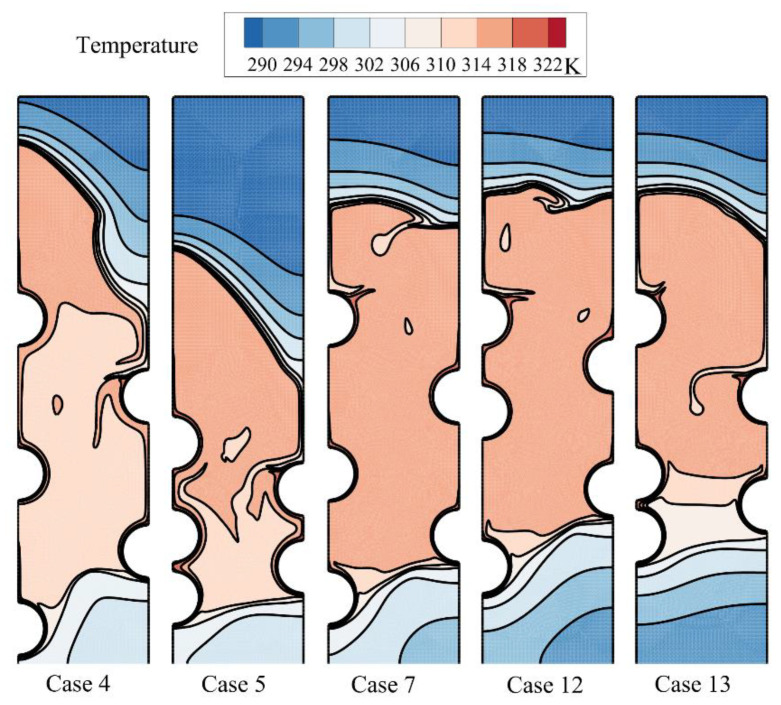
The isotherms for five different arrangements of the tubes after two hours of charging.

**Figure 10:**
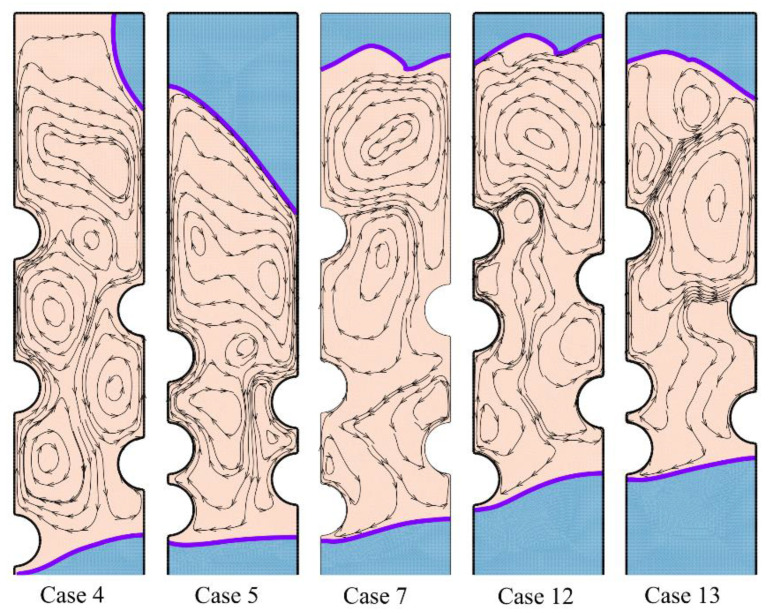
The streamlines and phase change interfaces for five different tubes’ arrangement after three hours of charging.

**Figure 11:**
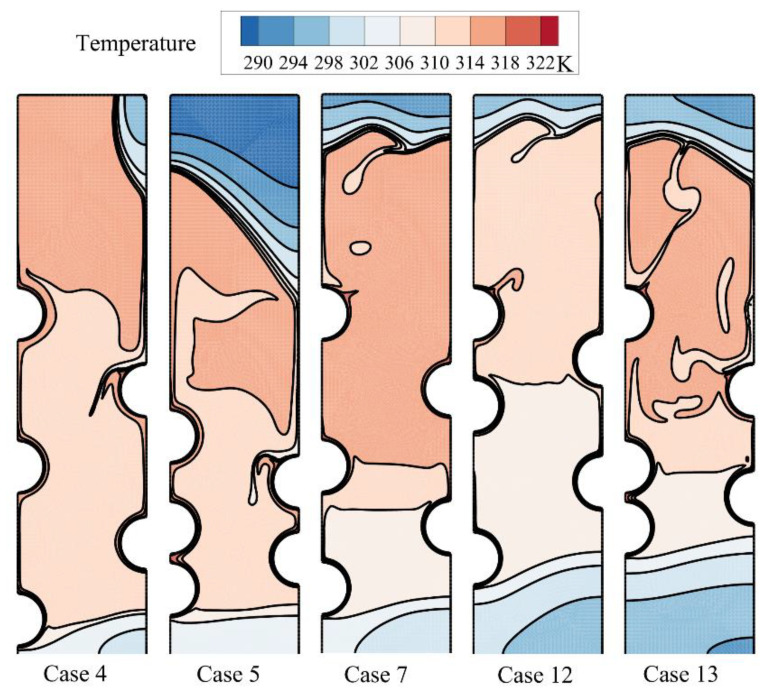
The isotherms for five different tubes’ arrangement after three hours of charging.

**Figure 12:**
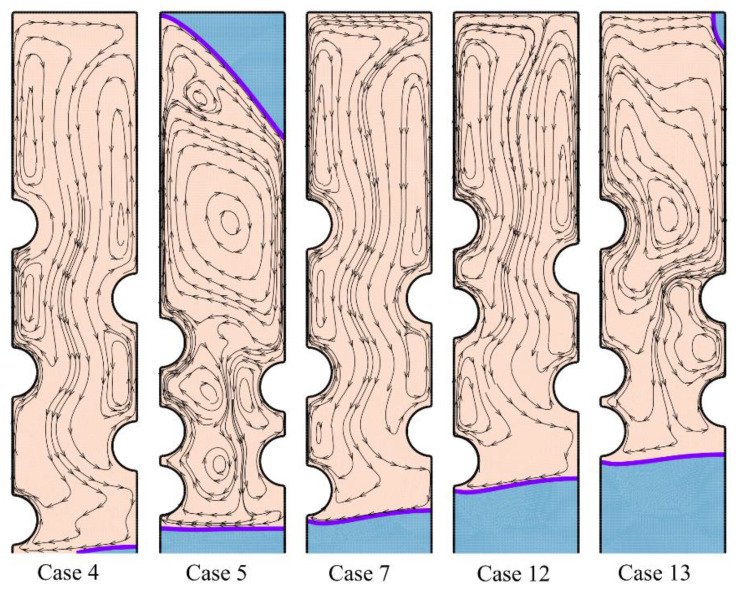
The streamlines and phase change interfaces for five different tubes’ arrangement after four hours of charging.

A systematic investigation of melting phase change heat transfer can be performed by analyzing streamlines and temperature distribution contours. Figure 6, Figure 7, Figure 8, Figure 9, Figure 10, Figure 11 and Figure 12 show the streamlines and isotherms for the optimum case (Case 4) and Cases 5, 7, 12, and 13. The results are reported every hour of charging time. The blue region in streamline figures denotes the solid PCM, and the light orange shows the liquid PCM. The thick line between these two regions is the melting interface line.

Figure 6 shows the streamlines just one hour after the commencement of thermal charging, and Figure 7 depicts the corresponding isotherms.

As time passes, the heat from HTF tubes accumulates in the solid PCM in the form of sensible heat, and the temperature of PCM increases around the tubes. As can be seen, the melting process starts around the hot tubes, advances to the space between the tubes and inclines toward the top regions. At this stage, it is clear that each tube develops a melting region around it. When the tubes are close to each other, the local melting regions reach each other and merge. Figure 7 displays hot regions around the tubes and cold regions far away from the tubes. The top regions of molten sites are also warmer due to the nature of buoyancy flows.

The melting process after two hours of charging time can be followed in Figure 8 and Figure 9.

The local regions around the HTF tubes merges, and there is a wide molten zone in the center of the enclosure. Still, the top and bottom zones are in a solid-state. As can be seen, the solid region at the bottom is mostly under the influence of the first row of tubes (*HL*_0_/*D*). This is while the location of other tubes does not show a significant impact on the bottom. In contrast, the second and third tube rows mostly impact the top melting interface. Shifting the tubes toward the top also shifts the melting interface upward. Figure 9 depicts that in all cases except the optimum case, the enclosure’s central region is at a high temperature. This is while for the optimum Case 4, only the top region is at a high temperature. This means that the melting rate is strong for the optimum case, and most of the HTF tubes’ heat is being consumed by the latent heat.

Figure 10 and Figure 11 show the same trend as Figure 8 and Figure 9.

The difference is that the molten region was further extended. At this stage, the solid regions were shrunk toward the bottom and the top. There is a notable difference between the optimum case and the other cases at this stage. Placing the first row of the HTF tubes at the bottom of the shell helps the shrinking of the solid region in the bottom part of the enclosure. The third row, placed at the highest location, melted most of the top regions. Figure 11 shows that the center regions of all other cases is still very hot while the top region of case 4 is hot. The hot temperature at the top contributes to the melting rate, while a lower temperature at central regions around the HFT tubes improves the heat transfer from the tubes due to large temperature differences.

**Figure 13:**
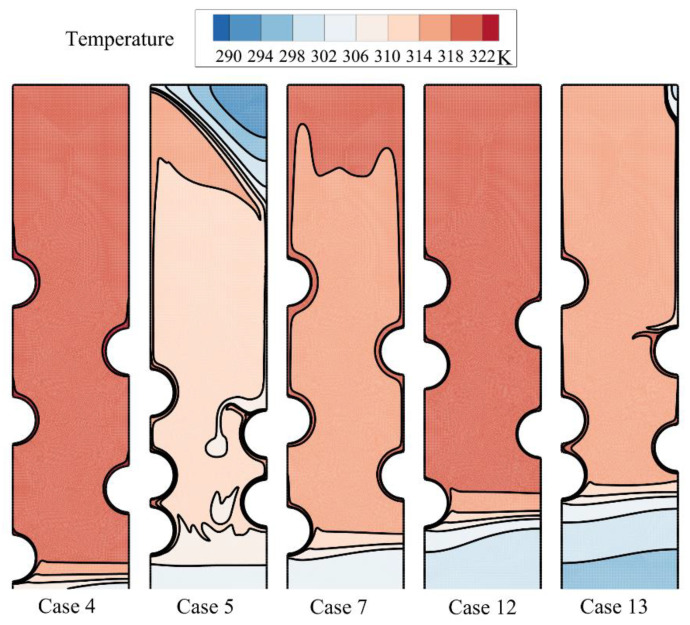
The isotherms for five different tubes’ arrangement after four hours of charging.

Figure 12 and Figure 13 show the final stage of melting in the enclosure.

In the optimum case 4, the top region completely melted down, and there is a negligible amount of the solid PCM at the bottom-right corner. Since there was no significant amount of the solid PCM to absorb the HTF heat in the form of latent heat, the temperature of the molten PCM is reaching the HFT temperature in most parts of the enclosure. The other cases mostly melted the PCM at the top region except case 5, in which the tubes were concentrated at the bottom. The attention to streamlines for this design shows that there are two separate circulation flows in the enclosure. Since the circulation flows are separated, the HTF heat cannot directly reach the top region and slow down the melting of the top zones. The amount of the solid PCM at the bottom is lower than in other cases (cases 7, 12, and 13), since the circulation flow at the bottom is injecting the heat into this region. The analysis on the final stage of melting for all cases showed that the placement of the first row toward the bottom was the most significant approach to melting down the bottom region. Moreover, the even distributions of the second and third rows in the enclosure helped with a general smooth circulation-free convection flow and the rapid melting of the top regions.

## 5. Conclusions

The melting thermal energy storage of an LHTES unit was addressed theoretically. The LHTES unit was modeled as a symmetric system consisting of five HTF tubes. The geometrical design of tube placement was optimized by employing the Taguchi method. The phase change behavior of the system was systematically investigated using LF graphs, streamlines, and isotherm contours. The main findings of the research can be summarized as follows:

- Based on the Taguchi design, the first row of the HTF tubes should be placed at the lowest possible point while the other tubes should be distributed evenly in the enclosure.
- The charging time of the LHTES unit could be changed by about 76% by just changing the location of tubes in the enclosure.
- From the streamlines and melting interfaces, it can be concluded that the formation of streamlines and free-convection flow circulation in each step of the melting process are the key points in the design of LHTES. Special attention should be paid to the streamline at the final stages of the charging process. A general uniform large circulation flow in the enclosure was much better than several small and week circulation flows.
- The Taguchi method could be used to effectively propose the optimum design of an LHTES unit with few simulations. Thus, this approach seems useful in the future design of energy storage systems.

## Figures and Tables

**Table 1:** Thermo-physical properties of RT35 [33].

Property	*ρ* (kg m^−3^)	*L_f_* (kJ kg^−1^)	*C_p_* (kJ kg^−1^ K)	*k* (W m^−1^ K)	*μ* (N s m^−2^)	*T_L_* (°C)	*T_S_* (°C)	*β* (1 K^−1^)
Values	815	170	2.0	0.2	0.023	35	29	0.0006

**Table 2:** The ranges and levels of the control parameters.

Factors	Description	Level 1	Level 2	Level 3	Level 4
**A**	*HL*_0_/*D* (height of the first tube)	1.2	1.8	2.4	3.0
**B**	*HL*_1_/*D* (height of the second tube)	1.2	1.8	2.4	3.0
**C**	*HL*_2_/*D* (height of the fourth tube)	1.2	1.8	2.4	3.0

**Table 3:** Taguchi L16 orthogonal table for three geometrical design parameters and four levels.

Experiment Number	Design Parameters			Time (s) for *LF* = 0.75	Signal-to-Noise (S/N) Ratio
	*HL*_0_/*D*	*HL*_1_/*D*	*HL*_2_/*D*		
**1**	1.2	1.2	1.2	13,633	−82.6918
**2**	1.2	1.8	1.8	13,613	−82.6791
**3**	1.2	2.4	2.4	9025	−79.1089
**4**	1.2	3.0	3.0	8205	−78.2816
**5**	1.8	1.2	1.8	11,977	−81.5670
**6**	1.8	1.8	1.2	13,850	−82.8290
**7**	1.8	2.4	3.0	9161	−79.2389
**8**	1.8	3.0	2.4	9206	−79.2814
**9**	2.4	1.2	2.4	14,742	−83.3711
**10**	2.4	1.8	3.0	9374	−79.4385
**11**	2.4	2.4	1.2	11,096	−80.9033
**12**	2.4	3.0	1.8	10,332	−80.2837
**13**	3.0	1.2	3.0	12,603	−82.0095
**14**	3.0	1.8	2.4	10,710	−80.5958
**15**	3.0	2.4	1.8	12,379	−81.8537
**16**	3.0	3.0	1.2	10,104	−80.0899

**Table 4:** The rank values of the control parameters based on the S/N ratio.

Level	*HL*_0_/*D*	*HL*_1_/*D*	*HL*_2_/*D*
1	−80.69	−82.41	−81.63
2	−80.73	−81.39	−81.60
3	−81.00	−80.28	−80.59
4	−81.14	−79.48	−79.74
Delta	0.45	2.93	1.89
Rank	3	1	2

**Table 5:** The best design for the minimum thermal charging time based on the Taguchi method.

Optimum Factors			Charging Time of *LF* = 0.75	
*HL*_0_/*D*	*HL*_1_/*D*	*HL*_2_/*D*	Taguchi Prediction	Tested Case
1.2	3.0	3.0	7915	8205

## Data Availability

Data is contained within the article.

## Nomenclature

*A_m_* (kg m^−3^ s^−1^)	mushy zone constant	*t_m_* (s)	melting/solidification time
*C_p_* (J kg^−1^ K^−1^)	specific heat	*T* (K)	temperature
*g* (ms^−2^)	gravity	*T_e_* (K)	mean temperature after the phase change process
*k* (W m^−1^ K^−1^)	thermal conductivity	*T_i_* (K)	initial temperature
*L_f_* (J kg^−1^)	latent heat of fusion		
*LF*	the normal total amount of melted phase change mateial		
*m* (kg)	mass	**Greek symbols**	
*P* (Pa)	pressure	*β* (K^−1^)	expansion coefficient
*Q* (J)	thermal storage/recovery capacity	*λ*	local liquid fraction
$\dot{Q}$ (W)	thermal storage/recovery rate	*μ* (kg m^−1^ s^−1^)	dynamic Viscosity
*T_L_* (K)	liquidus temperature	*ρ* (kg m^−3^)	density
*T_s_* (K)	solidus temperature	Δ*H* (J kg^−1^)	latent heat of fusion
$\vec{V}$ (m s^−1^)	velocity		
